# Prescription Digital Therapeutics for Substance Use Disorder in Primary Care: Mixed Methods Evaluation of a Pilot Implementation Study

**DOI:** 10.2196/59088

**Published:** 2024-09-02

**Authors:** Jessica Mogk, Abisola E Idu, Jennifer F Bobb, Dustin Key, Edwin S Wong, Lorella Palazzo, Kelsey Stefanik-Guizlo, Deborah King, Tara Beatty, Caitlin N Dorsey, Ryan M Caldeiro, Angela Garza McWethy, Joseph E Glass

**Affiliations:** 1 Kaiser Permanente Washington Health Research Institute Seattle, WA United States; 2 Department of Health Systems and Population Health School of Public Health University of Washington Seattle, WA United States; 3 Mental Health and Wellness Services Kaiser Permanente Washington Renton, WA United States; 4 Mental Health and Wellness Kaiser Permanente Oakland, CA United States

**Keywords:** implementation, digital therapeutics, substance use disorder, practice facilitation, health coaching, primary care, mobile phone

## Abstract

**Background:**

Delivering prescription digital therapeutics (ie, evidence-based interventions designed to treat, manage, or prevent disorders via websites or smartphone apps) in primary care could increase patient access to substance use disorder (SUD) treatments. However, the optimal approach to implementing prescription digital therapeutics in primary care remains unknown.

**Objective:**

This pilot study is a precursor to a larger trial designed to test whether implementation strategies (practice facilitation [PF] and health coaching [HC]) improve the delivery of prescription digital therapeutics for SUDs in primary care. This mixed methods study describes outcomes among patients in the 2 pilot clinics and presents qualitative findings on implementation.

**Methods:**

From February 10 to August 6, 2021, a total of 3 mental health specialists embedded in 2 primary care practices of the same integrated health system were tasked with offering app-based prescription digital therapeutics to patients with SUD. In the first half of the pilot, implementation activities included training and supportive tools. PF (at 1 clinic) and HC (at 2 clinics) were added in the second half. All study analyses relied on secondary data, including electronic health records and digital therapeutic vendor data. Primary outcomes were the proportion of patients reached by the prescription digital therapeutics and fidelity related to ideal use. We used qualitative methods to assess the adherence to planned activities and the barriers and facilitators to implementing prescription digital therapeutics.

**Results:**

Of all 18 patients prescribed the apps, 10 (56%) downloaded the app and activated their prescription, and 8 (44%) completed at least 1 module of content. Patients who activated the app completed 1 module per week on average. *Ideal use* (fidelity) was defined as completing 4 modules per week and having a monthly SUD-related visit; 1 (6%) patient met these criteria for 10 weeks (of the 12-week prescription period). A total of 5 (28%) patients had prescriptions while HC was available, 2 (11%) were successfully contacted, and both declined coaching. Clinicians reported competing clinical priorities, technical challenges, and logistically complex workflows in part because the apps required a prescription. Some pilot activities were impacted by staff turnover that coincided with the COVID-19 pandemic. The facilitators to implementation were high engagement and the perception that the apps could meet patient needs.

**Conclusions:**

The pilot study encountered the barriers to implementing prescription digital therapeutics in a real-world primary care setting, especially staffing shortages, turnover, and competing priorities for clinic teams. The larger randomized trial will clarify the extent to which PF and HC improve the implementation of digital therapeutics.

**Trial Registration:**

ClinicalTrials.gov NCT04907045; https://clinicaltrials.gov/study/NCT04907045

## Introduction

### Background

Substance use disorders (SUDs) are very prevalent, with >1 in 6 (17%) Americans aged ≥12 years reported experiencing a past-year SUD in 2022 [1]. Despite known negative health effects, such as mental health comorbidities, disability, and death, SUDs often go untreated [2-4]. Patients are open to receiving SUD treatment in primary care [5]; however, the services and capacity are not available to meet demand [6-10].

Digital interventions have the potential to increase patient access to SUD treatment. One specific form of digital intervention includes those that require a prescription and are designed to treat, manage, or prevent disorders (ie, prescription digital therapeutics) [11,12]. Prescription digital therapeutics may deliver behavioral health interventions such as assessments, educational content, and activities to help patients change their thoughts and behaviors. Because they require a prescription, they are often used in conjunction with other treatments delivered by a clinician (eg, counseling or medication) [11,13]. More broadly, digital interventions for SUD are acceptable to patients [14,15] and can improve outcomes in real-world settings [16-19]. There is also evidence demonstrating that digital interventions can reduce the amount of time clinicians need to spend with patients during the course of SUD treatment [20,21].

Primary care is an ideal setting to implement prescription digital therapeutics for SUD because screening and intervention for substance use in primary care is already recommended by the US Preventive Services Task Force [22,23], which creates a mechanism for identifying many people who may be in need of treatment. Furthermore, most people with SUD prefer to receive treatment in primary care [5]. Prescription digital therapeutics are a potentially low-barrier mode of treatment intervention because patients can engage in them via the comfort of home. Despite their potential to improve patient care, prior research has demonstrated that there are many barriers to the implementation of digital interventions [24], and we are aware of no implementation studies on prescription digital therapeutics, specifically. Little data are available about workflows and implementation strategies that can be used by health care systems to integrate these interventions into primary care practices.

### Objectives

This study is a precursor to the Digital Therapeutics for Opioids and Other SUDs (DIGITS) Trial, which is designed to test whether implementation strategies can increase and improve the delivery of digital therapeutics for SUDs (trial registration: ClinicalTrials.gov NCT05160233) [25]. Here, we present the results of the DIGITS Trial pilot study, in which digital therapeutics for SUDs were implemented in 2 primary care clinics in an integrated health system (trial pilot registration: ClinicalTrials.gov NCT04907045). In this study, mental health specialists (licensed independent clinical social workers [LICSWs]) embedded in primary care practices were tasked with determining patient eligibility to be prescribed the apps, offering the apps to patients, and executing workflows that enable the provision of the apps. We note that in the case of prescription digital therapeutics in the United States, clinicians must determine whether patients meet strict clinical eligibility criteria before issuing a prescription, and this information is communicated by the app vendor in the form of a “label” that has been authorized by the US Food and Drug Administration (FDA).

In this study, we quantitatively examined the implementation during the pilot study by describing the proportion of eligible patients reached by the digital therapeutics and fidelity related to ideal use. We also present qualitative results from a formative evaluation that explored the barriers and facilitators to implementation within the study context. This study was designed to produce data that help health systems understand the potential benefit of strategic approaches to increase the uptake of digital interventions for SUDs and prioritize strategies for increasing access to care for SUDs.

## Methods

### Setting

The DIGITS pilot study took place at Kaiser Permanente Washington (KPWA), a large integrated health system in Washington State. We selected 2 primary care clinics (referred to as masked clinics A and B) in Seattle, Washington, for the pilot, on the basis of the presence of highly skilled LICSWs willing to participate in a pilot study of digital therapeutics. We also intentionally selected clinics of disparate sizes. Clinic A was a large outpatient medical center with approximately 29,000 primary care empaneled patients. Clinic B was a newer and smaller medical center with approximately 2500 empaneled patients.

### Ethical Considerations

In this implementation study, clinical and implementation decisions were enacted by clinicians and health system leaders of KPWA. Research activities were reviewed by the KPWA Institutional Review Board, which granted ethical approvals including waivers of consent and Health Insurance Portability and Accountability Act authorization (1794767-2).

### Purpose of the Pilot

The DIGITS pilot study was conducted to evaluate the feasibility of integrating digital therapeutics into primary care and to prepare for the DIGITS Trial, an implementation trial funded by the US National Institute on Drug Abuse. The DIGITS Trial was designed to evaluate whether practice facilitation (PF) and health coaching (HC) can improve the implementation of digital therapeutics for SUDs in primary care when provided in addition to standard implementation strategies in a 2 × 2 factorial design [25].

Standard implementation involved a 2-hour training; quality improvement meetings; an implementation toolkit consisting of clinician workflow aids, patient pamphlets, and scripts to help clinicians introduce the digital therapeutic to patients; and electronic health record (EHR) tools including documentation (ie, charting) templates and after-visit summaries, an order set, and a population management workbench (ie, EHR registry). Standard implementation was based on the implementation strategies previously used by clinical leaders at KPWA to implement digital interventions for depression and anxiety (these were the apps that did not require prescriptions) [26,27]. We adapted the original standard implementation approach on the basis of the learnings from 2 user-centered design studies focused on SUD apps [28,29] and made further refinements to accommodate the novel constraints introduced by prescription digital therapeutics.

PF is an evidence-based clinician-facing implementation strategy designed to support clinicians in overcoming implementation and workflow challenges [30,31]. Previous studies have used PF to support the implementation of SUD treatments [32-34]. HC is a patient-facing strategy designed to support patient self-management and engagement in treatment [35]. Previous studies have demonstrated various forms of coaching to be effective for engaging patients in digital therapeutics [36-40]. For our trial, we developed a digital therapeutic HC protocol designed for use by a medical assistant (MA).

**Figure 1:**
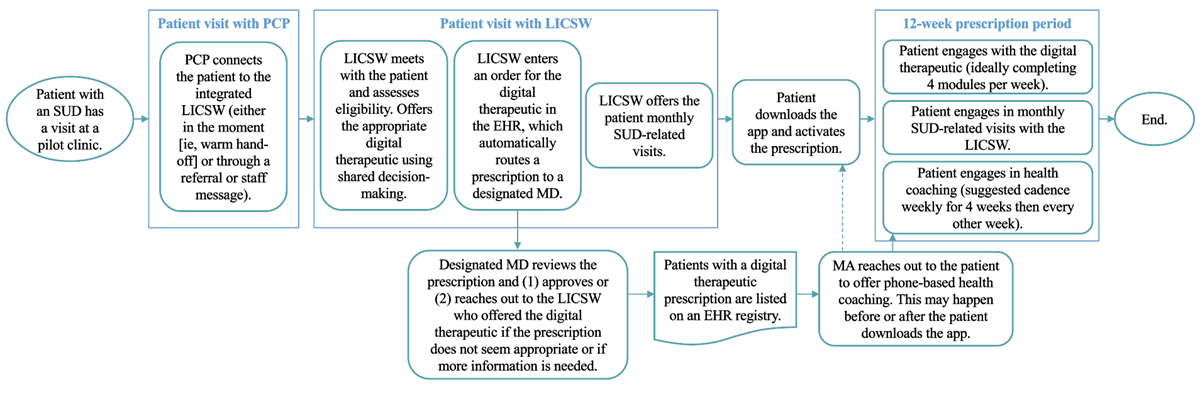
Workflow for connecting patients to digital therapeutics and supporting them to engage in substance use disorder (SUD) treatment. Workflow assumes the patient is interested in SUD treatment in general and is open to trying a digital therapeutic. A patient can opt out at any stage of the process (eg, decide not to get a digital therapeutic prescription) or can opt out of certain parts of the intervention (eg, engage with the digital therapeutic but opt not to schedule SUD-related visits with the licensed independent clinical social worker [LICSW; these staff members are embedded in primary care]). EHR: electronic health record; MA: medical assistant (this staff member is centralized); MD: medical doctor; PCP: primary care provider.

The digital therapeutics implemented were reSET and reSET-O, prescription smartphone apps made by Pear Therapeutics, which had been authorized by the FDA for the treatment of SUDs (not including alcohol use disorders [AUDs] unless they are accompanied by drug use) and opioid use disorder (only when accompanied by prescription buprenorphine use), respectively. reSET is a 90-day prescription, and reSET-O is an 84-day prescription. Both apps leveraged cognitive-behavioral approaches and are shown to improve patient outcomes in randomized controlled trials conducted in specialty addiction treatment settings [18,20,21,41-44]. The main therapeutic content of reSET and reSET-O is delivered in 31 “core modules” within the apps. In addition, the apps incorporate contingency management operationalized as monetary incentives in the form of gift cards to incent negative drug screens and completion of learning modules. Digital therapeutic prescriptions were entered into the EHR by LICSWs and routed to a medical doctor for approval. The workflow for connecting patients to the digital therapeutics and supporting them to engage in SUD treatment is displayed in Figure 1.

### Timeline and Population

Pilot activities involved 2 distinct 3-month periods. During the first period (from February 10 to May 5, 2021), 1 LICSW from each of the 2 pilot clinics was trained to offer reSET and reSET-O. During this time, LICSWs offered the apps to patients in the absence of PF or HC. This supported our goal of testing the pilot standard implementation materials. During the second period (from May 6 to August 6, 2021), clinic A received both PF and HC, and clinic B received HC only.

The study’s quantitative outcome evaluation was consistent with pragmatic trial principles [45], entirely relying on secondary data sources. We report on two analytic cohorts: (1) a “main analytic cohort” consisted of patients who met predefined eligibility criteria, allowing us to calculate the reach of the digital therapeutic in a defined patient population (which was consistent with the main trial eligibility criteria) and (2) a secondary “prescribed cohort,” which consisted of all patients who were prescribed the digital therapeutic, regardless of whether they met criteria for the analytic cohort.

The main analytic cohort consisted of patients aged ≥18 years who had a primary care visit at clinic A or B during the accrual period (defined in the *Data Collection and Analysis* section) and had a high-scoring screen for drug use on the day of the visit or at any time in the prior year. A *high-scoring screen* was defined as a score of 4 on the Single-Item Screen for Cannabis [46] (the highest score, indicating daily or almost daily cannabis use), a score >0 for other drugs on the Single-Item Screen for Drugs [47,48] (indicating any past-year use of other drugs), or both. Patients who have requested through the health system not to be contacted for research or not to have their chart reviewed for research were excluded. We included patients who had high-scoring substance use screens rather than patients with documentation of an active SUD diagnosis in analyses to avoid the potential for identification bias [49]. A large proportion of patients in health care settings who have an SUD do not receive a diagnosis [50], and our implementation activities could have increased SUD diagnoses (eg, clinicians may have added SUD diagnoses in the process of offering digital therapeutics for SUD treatment, which would bias the study denominator if it was defined by SUD diagnosis). In contrast, most patients in KPWA (approximately 90%) receive annual SUD screening [50], and our intervention was unlikely to change which patients were screened, so our chosen denominator was more likely to be free of identification bias.

The second cohort, the prescribed cohort, consisted of patients who were prescribed the digital therapeutics. Patients in this cohort did not necessarily meet the criteria for the analytic cohort (eg, they may not have had a high-scoring drug screen or a visit at clinic A or B during the accrual period or before their prescription) but were determined by clinicians to meet eligibility criteria for one of the digital therapeutics. For example, a patient who indicated on the Single-Item Screen for Cannabis that they used cannabis weekly (as opposed to daily) would not have met the criteria for the analytic cohort, but a clinical discussion may have revealed a need for SUD treatment.

### Data Collection and Analysis

**Figure 2:**
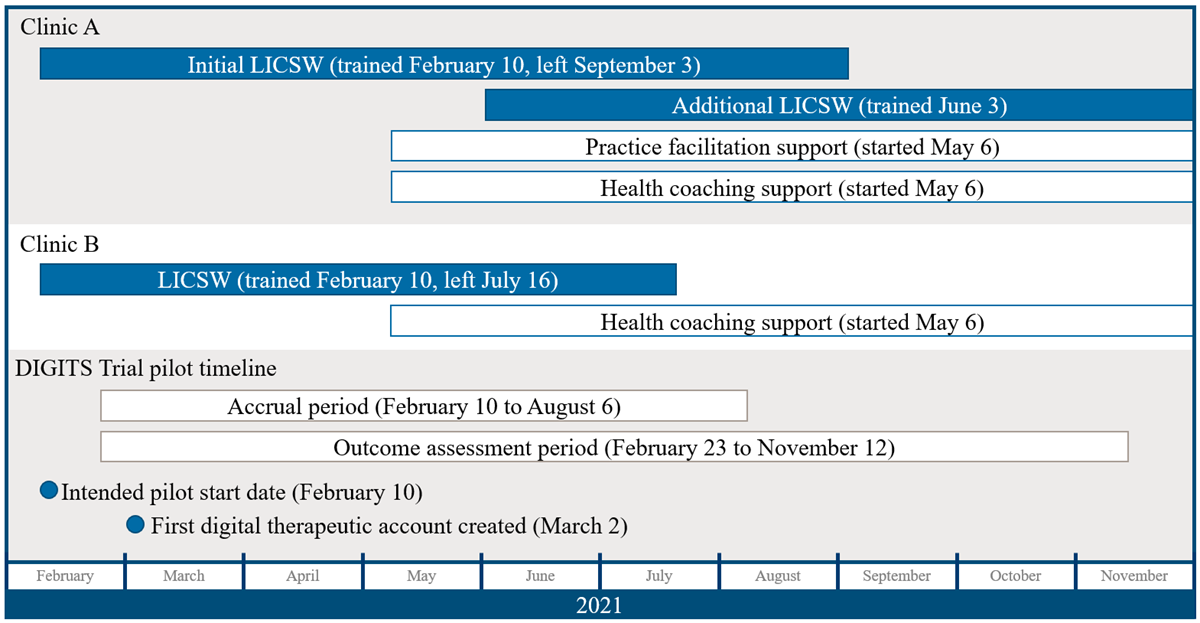
Digital Therapeutics for Opioids and Other Substance Use Disorders (DIGITS) pilot study timeline. The pilot started with a standard implementation period, in which 1 licensed independent clinical social worker (LICSW) from each of the 2 pilot clinics was trained to offer reSET and reSET-O. The pilot launch was delayed due to technical challenges, and the first patient accounts for the digital therapeutic were created about 3 weeks after LICSWs were trained. Practice facilitation support (for clinic A) and health coaching (for both clinics) were added. During this period, an additional LICSW was trained at clinic A, and the initial LICSWs at both clinics left the organization.

Data were collected from the EHR for an accrual period and an outcomes assessment period (see Figure 2 for the DIGITS Trial pilot timeline). The intended launch date was February 10, 2021 (see the *Timeline and Population* section), but technical problems delayed reSET and reSET-O account creation and prescriptions until March 2, 2021. Specifically, clinicians could not access the vendor’s website from web browser versions that were installed in clinical offices; to resolve this, the app vendor ultimately modified their website. In addition, the EHR order set did not initially route prescriptions to physicians, which required EHR programmer intervention. We set a patient accrual period from February 23 to August 6, 2021, which captures everyone who was prescribed the digital therapeutics, including patients who may have visited clinics slightly before the technical problems were resolved. The outcomes assessment period (February 23 to November 12, 2021) included the accrual period (since the outcome of reach of the digital therapeutic, defined below, could happen as early as the day of eligibility); a 1-week grace period (to allow patients who became eligible on the last day of the accrual period to have at least 1 week to be reached); and 3 follow-up months to capture app use for patients who had a 12-week digital therapeutic prescription at any point during the pilot.

Baseline characteristics from the EHR included demographics, address, insurance status, health care use, and mental health and SUD screening information and diagnoses. For patients having multiple eligible visits (those who were aged ≥18 years and had a high-scoring screen), we used the first eligible visit as the “qualifying visit” on which to anchor time-varying covariates and outcomes. For patients who were prescribed the digital therapeutics but did not have an eligible visit, we used the prescription date as the qualifying visit. The time period for constructing baseline clinical characteristics was from 2 years before until the day before the qualifying visit. Age was measured on the date of the qualifying visit. Sex (binary male or female), race, and ethnicity were from the time of the data pull to acquire the most current data. Insurance and address were measured on the date of or in the month before the qualifying visit, depending on data availability. Rurality and urbanity was defined using the 2010 rural-urban commuting area codes and 2010 Census Tract geocoding [51].

Our primary outcomes were clinic-level measures of reach and fidelity [52]. *Reach* was defined as the number and proportion of patients who were prescribed reSET or reSET-O, downloaded the app, activated their prescription, and completed at least 1 module within the app. *Fidelity* was defined as the average (across patients) of the number of weeks (out of 12 possible weeks) in which patients completed at least 4 modules per week and had a past-month SUD-related visit. This definition of fidelity is based on Pear Therapeutic’s recommendation that patients complete 4 modules per week (the maximum number of modules per week for which a patient can receive a contingency management reward) and the FDA label requirement and clinical recommendation that patients use the digital therapeutic while under the care of a clinician (our health system leaders–determined monthly SUD-related visits satisfied this requirement) [20,44,53,54]. We used a database provided by the vendor to obtain the data related to app use such as activations and module completion during the outcomes assessment period.

Additional outcomes were treatment engagement and additional reach and fidelity measures. Treatment engagement was defined as the average (across patients) of the number of months in which patients had at least 1 visit for SUD treatment. Additional reach measures (which were subcomponents of the primary reach outcome measure) were the number and proportion of patients who were prescribed reSET or reSET-O (regardless of whether they downloaded the app and activated their prescription) and the number and proportion of patients who were prescribed the app and subsequently downloaded it and activated their prescription. Additional fidelity measures were the average number of weeks (out of 12 possible weeks) in which patients completed at least 1 module per week or completed at least 4 modules per week. Treatment engagement and fidelity-3 were subcomponents of the primary fidelity outcome measure. Adoption was measured to record if clinicians other than LICSWs completed training to prescribe the digital therapeutics. Outcome definitions are presented in Table 1.

**Table 1:** Digital Therapeutics for Opioids and Other SUDs^a^ pilot study outcome measures.

Measure			Definition
**Primary outcomes**			
	Reach	The number and proportion of patients who were prescribed reSET or reSET-O, downloaded the app and activated their prescription, and completed at least 1 module within the app	
	Fidelity	The average of the sum of the number of weeks (out of 12 possible weeks) in which patients completed at least 4 modules per week and had a past-month SUD-related visit	
**Additional prespecified outcomes**			
	Treatment engagement	The average of the sum of the number of months in which patients had at least 1 visit for SUD treatment	
	Reach-2	The number and proportion of patients who were prescribed reSET or reSET-O	
	Reach-3	The number and proportion of patients who were prescribed reSET or reSET-O, downloaded the app, and activated their prescription	
	Fidelity-2	The average of the sum of the number of weeks (out of 12 possible weeks) in which patients completed at least 1 module per week	
	Fidelity-3	The average of the sum of the number of weeks (out of 12 possible weeks) in which patients completed at least 4 modules per week	
	Adoption	The proportion of other health care providers initiating a patient on reSET or reSET-O, overall and by provider type	

^a^SUD: substance use disorder.

**Figure 3:**
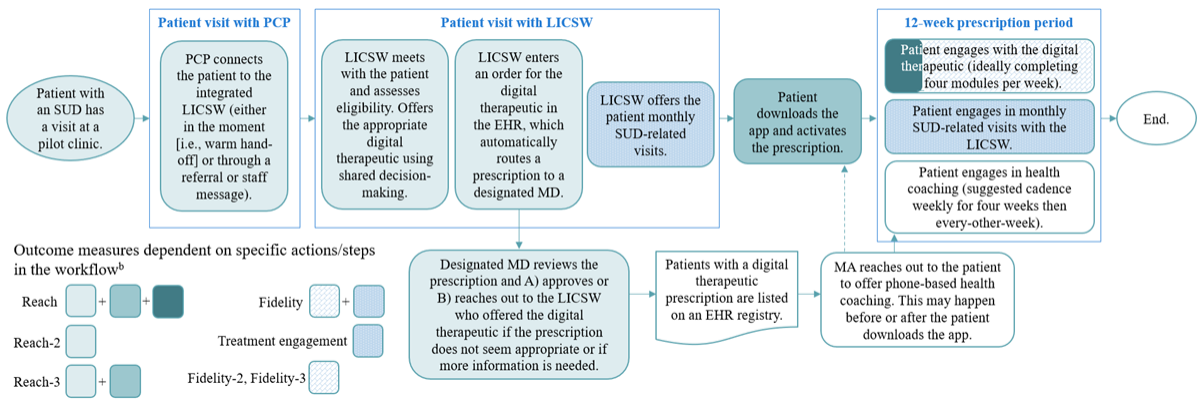
How staff and patient activation contributes to reach and fidelity in the Digital Therapeutics for Opioids and Other Substance Use Disorders (DIGITS) pilot study. Outcomes superimposed on the workflow to highlight who needs to act to achieve each outcome. Workflow for connecting patients to the digital therapeutics and supporting them to engage in substance use disorder (SUD) treatment assumes the patient is interested in SUD treatment in general and open to trying a digital therapeutic. A patient can opt out at any stage of the process (eg, decide not to get a digital therapeutic prescription) or can opt out of certain parts of the intervention (eg, engage with the digital therapeutic but opt not to schedule SUD-related visits with the LICSW). ^b^Reach: the number and proportion of patients who were prescribed reSET or reSET-O, downloaded the app and activated their prescription, and completed at least one module within the app. Fidelity: the average of the sum of number of weeks (out of 12 possible weeks) in which patients completed at least four modules per week and had a past-month SUD-related visit. Treatment engagement : the average of the sum of the number of months in which patients had at least one visit for SUD treatment. Reach-2: the number and proportion of patients who were prescribed reSET or reSET-O. Reach-3: the number and proportion of patients who were prescribed reSET or reSET-O, downloaded the app and activated their prescription. Fidelity-2: the average of the sum of number of weeks (out of 12 possible weeks) in which patients completed at least one module per week. Fidelity-3: the average of the sum of number of weeks (out of 12 possible weeks) in which patients completed at least four modules per week. EHR: electronic health record; LICSW: Licensed Independent Clinical Social Worker (these staff members are embedded in primary care); MA: medical assistant (this staff member is centralized); MD: medical doctor; PCP: primary care provider.

While both of our primary outcomes required clinician and patient involvement, reach relied more heavily on the actions from clinicians, and fidelity relied more heavily on the actions from patients. For example, primary care providers (PCPs) and LICSWs were responsible for identifying the patients who were eligible for the digital therapeutics; PCPs would connect potentially eligible patients to the integrated LICSW, who would offer the digital therapeutics to patients. If the patient agreed to try the app and the designated medical doctor approved the prescription, that patient would be included in the numerator of reach-2. Downloading the digital therapeutic and activating the prescription (reach-3) was the responsibility of patients, although a motivated clinician with time to do so could assist with this step. Module completion (which was factored into reach, fidelity, fidelity-2, and fidelity-3 [module completion is critical because it is the primary way patients receive treatment from reSET or reSET-O]) required activation from patients, although clinicians were instructed to encourage patients to complete the modules. Treatment engagement (a subcomponent of fidelity) was the joint responsibility of clinicians and patients. Many external factors could influence treatment engagement (eg, appointment or scheduling access, patient ability to pay for SUD-related visits, etc). A visualization of outcomes superimposed on the implementation workflow to highlight who needs to act to achieve each outcome is included in Figure 3.

Data analysis was largely descriptive. Patient characteristics were described among eligible patients in clinics A and B and in the overall sample. Outcomes were calculated among the eligible sample, overall and separately within each of the 2 clinics. We additionally calculated outcomes among the secondary sample of patients who were prescribed reSET (including some patients who did not meet the eligibility criteria [eg, those who were prescribed reSET but did not have a high-scoring screen within a year before a primary care visit]) within each of the 2 clinics and in the overall sample.

Designated evaluators (LP and CND) conducted a formative evaluation grounded in the Dynamic Sustainability Framework [55,56] and the Framework for Reporting Adaptations and Modifications to Evidence-based Implementation Strategies [57,58]. The evaluation was focused on pilot successes, barriers, facilitators, implementation strategies, and adaptations made during implementation. Qualitative data were collected from training sessions, study team meetings, PF meetings, and meetings with primary care and mental health leaders using templated forms. Data were rapidly analyzed using an analysis matrix with predetermined domains [57,59,60] to capture the lessons learned from the DIGITS pilot and inform the main trial [61,62]. Additional details regarding the formative evaluation methods (including the templated forms and analysis matrix) are available in a report describing the trial protocol [25].

## Results

### DIGITS Pilot Study Activities

The DIGITS pilot study timeline and activities were largely carried out as planned, although staffing changes caused inconsistency in the availability of the digital therapeutics for patients who were otherwise eligible. At both clinics, the initial LICSW left the organization during the pilot (Figure 2). In clinic A, an additional (newly hired) LICSW was trained to offer reSET and reSET-O about 3 months after the pilot started. The initial LICSW from clinic A left the organization a few months after that. The second LICSW from clinic A was available to offer the digital therapeutics during this time. In clinic B, the LICSW left KPWA in the final month of the accrual period, and no other clinicians were immediately available at the clinic to offer the digital therapeutics. PF and HC were launched when planned.

### Implementation Strategy Delivery

#### Engagement in Standard Implementation

In 4 quality improvement meetings, JEG facilitated a review of recent app enrollment and engagement data. The LICSWs were prompted to share patient stories, and the mental health leader facilitated a discussion to gather information on the successes and challenges. This was a basis for conducting cyclical small tests of change and obtaining feedback to help improve implementation tools, such as the EHR templates, training materials, and job aids.

#### Engagement in PF

Experienced practice facilitators led 6 meetings for clinic A during the second period of the pilot: a one-on-one meeting with each of the 2 LICSWs; a meeting with clinic leaders; a meeting with both LICSWs together; a meeting with a PCP; and a meeting with the full implementation team (both LICSWs, the PCP, and the MA who usually worked with the PCP).

During one-on-one and leadership meetings, facilitators introduced the digital therapeutics, the DIGITS Trial, and PF; these meetings lasted 20 to 30 minutes. During group meetings, the facilitators shared audit and feedback reports that displayed LICSW visits with potentially eligible patients, reSET and reSET-O offers and account activations, and module completion and LICSW visits (these were typically SUD-related visits, which would qualify as treatment engagement) for patients who had a prescription and activated the app. Teams discussed the barriers and facilitators to implementation, brainstormed ideas for improving reach and fidelity, and carried out plan-do-study-act (PDSA) cycles. Examples of PDSA cycles completed include adding new members to the implementation team, creating a list of reSET and reSET-O EHR shortcuts for quick reference, and educating PCPs about the digital therapeutics. The 2 group meetings lasted 55 minutes each.

#### Engagement in HC

The MA offered HC to 4 (80%) of 5 patients who had a digital therapeutic prescription in the second period of the pilot. The fifth patient was deemed ineligible for HC by the LICSW who offered them the app (reason not documented). The HC MA reached out to patients up to 5 times using phone calls and secure messages in the EHR. The HC did not receive responses from 2 (50%) patients. Of the 2 who could be reached, 1 declined because they were transitioning to inpatient SUD treatment, and the other was not interested in additional support.

The HC outreach call consisted of introductions; agenda setting; orientation to the HC role (encouragement, support behavior change, and goal setting, help with problem-solving and overcoming barriers); discussion of the patient experience with the digital therapeutic so far (eg, downloading the app, using the access code, completing modules); reinforcing the goal of completing 4 modules each week; and problem-solving as needed. The MA was trained in motivational interviewing and HC and focused on building positive, trusting relationships with patients. While the HC strategy was designed to involve weekly phone outreach for the first 4 weeks of the digital therapeutic prescription and secure messages in the EHR every other week, after that, the MA was prepared to adapt HC to fit patient preferences and needs.

For approximately 1 month of the pilot, the MA piloted another outreach method of using an existing patient registry to identify patients with SUD who were potentially eligible for the digital therapeutics. The HC would send a secure message to the patient to ask if they might be interested in scheduling a visit with an LICSW to discuss the digital therapeutics. Among the 1552 patients in the main analytic cohort, 23 (1.48%) patients were sent a secure message, and 5 (0.32%) patients messaged back. No additional prescriptions resulted from this strategy.

### Quantitative Results

#### Patients Eligible for Quantitative Analyses

**Figure 4:**
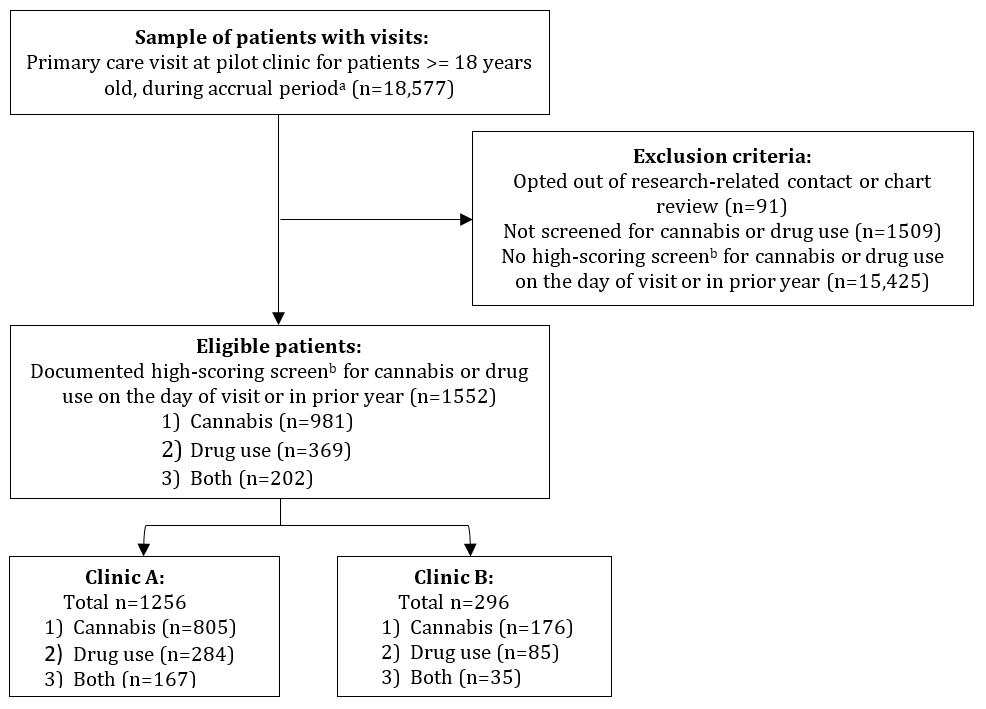
Identification of eligible patients for the Digital Therapeutics for Opioids and Other Substance Use Disorders (DIGITS) pilot study. The DIGITS pilot study used mixed methods to evaluate the implementation of 2 digital therapeutics (reSET and reSET-O) for substance use disorders in 2 primary care clinics in the same integrated health system in Washington State. ^a^The accrual period was from February 23, 2021, to August 6, 2021. ^b^A high-scoring screen was defined as a score of 4 (the highest score) on the Single-Item Screen for Cannabis (indicating daily or almost daily cannabis use) and a score >0 for other drugs on the Single-Item Screen for Drugs (indicating any other drug use).

During the accrual period, 18,577 patients had visited clinic A or B. Of them, 1552 (8.35%) patients had a documented high-scoring drug screen (clinic A: n=1256, 80.93%; clinic B: n=296, 19.07%; Figure 4).

Patients (n=1552) included in the main analytic cohort were aged 38.8 (SD 15.6) years on average and generally younger in clinic B than in clinic A (Table 2). About half of the patients (n=795, 51.22%) were female. The main analytic cohort was mostly White (n=1095, 70.55%); the next most common race categories were Asian (n=92, 5.93%), Black or African American (n=71, 4.57%), missing race data (n=141, 9.09%), and multiracial (n=73, 4.7%). Approximately 7.35% (n=114) of the patients were Hispanic, and 24.23% (n=376) of the patients were missing ethnicity data. Nearly every patient (n=1551, 99.9%) had an address in an urban Census Tract. Most patients had commercial insurance (clinic A: 579/1256, 46.1%; clinic B: 174/296, 58.8%); Medicare was the next most common insurance (clinic A: 161/1256, 12.82%; clinic B: 10/296, 3.4%). In addition, 47.49% (n=737) of patients had an anxiety diagnosis, and 39.56% (n=614) had a depression diagnosis. Most patients (cannabis: n=917, 71.8%; other drugs: n=435, 34.1%) had a history of high-scoring drug use screens before their qualifying visit, whereas only a small percentage had a documented SUD diagnosis (eg, <6% for each type of SUD). Only 11% (n=173) of patients were engaged in SUD-related care.

**Table 2:** Baseline characteristics of patients in the main analytic cohort.

Demographics				Clinic A: standard implementation, practice facilitation, and health coaching (n=1256)		Clinic B: standard implementation and health coaching (n=296)		Total (n=1552)
Age (y), mean (SD)				40.2 (16.2)		32.9 (10.9)		38.8 (15.6)
**Age group (y), n (%)**								
	18-24		182 (14.49)		60 (20.27)		242 (15.59)	
	25-34		459 (36.54)		153 (51.69)		612 (39.43)	
	35-44		218 (17.36)		48 (16.22)		266 (17.14)	
	45-54		126 (10.03)		13 (4.39)		139 (8.96)	
	≥55		271 (21.58)		22 (7.43)		293 (18.88)	
**Sex, n (%)**								
	Female		635 (50.56)		160 (54.05)		795 (51.22)	
**Race, n (%)**								
	Asian		67 (5.33)		25 (8.45)		92 (5.93)	
	Black or African American		54 (4.30)		17 (5.74)		71 (4.57)	
	American Indian or Alaska Native		—^a^		—		18 (1.16)	
	Native Hawaiian or Pacific Islander		—		—		5 (0.32)	
	White		916 (72.93)		179 (60.47)		1095 (70.55)	
	Multiple race		54 (4.30)		19 (6.42)		73 (4.70)	
	Other race		45 (3.58)		12 (4.05)		57 (3.67)	
	Missing race data		100 (7.96)		41 (13.85)		141 (9.09)	
**Ethnicity, n (%)**								
	Hispanic		89 (7.09)		25 (8.45)		114 (7.35)	
	Non-Hispanic		899 (71.58)		163 (55.07)		1062 (68.43)	
	Missing ethnicity data		268 (21.34)		108 (36.49)		376 (24.23)	
**Rurality of residence (Census Tract), n (%)**								
	Urban		1255 (99.92)		296 (100.00)		1551 (99.94)	
**Insurance type, n (%)**								
	Medicaid		38 (3.03)		12 (4.05)		50 (3.22)	
	Medicare		161 (12.82)		10 (3.38)		171 (11.02)	
	State subsidized		96 (7.64)		22 (7.43)		118 (7.60)	
	Private pay, self-funded, high deductible, and basic health		308 (24.52)		61 (20.61)		369 (23.78)	
	Commercial		579 (46.10)		174 (58.78)		753 (48.52)	
	Unknown		74 (5.89)		17 (5.74)		91 (5.86)	
≥1 year of health insurance enrollment, n (%)				953 (75.88)		193 (65.20)		1146 (73.84)
**Health status, n (%)**								
	**Mental health diagnoses**							
		Anxiety	594 (47.29)		143 (48.31)		737 (47.49)	
		Depression	493 (39.25)		121 (40.88)		614 (39.56)	
		Serious mental illness^b^	63 (5.02)		15 (5.07)		78 (5.03)	
	Positive screen for depression^c^		440 (46.56)		120 (54.30)		560 (48.03)	
	Any emergency visits		—		—		19 (1.22)	
	Any hospitalization		—		—		29 (1.87)	
**SUD^d^-related characteristics, n (%)**								
	**High-scoring drug use screens^e^**							
		Alcohol	132 (12.70)		29 (12.13)		161 (12.60)	
		Cannabis	757 (72.86)		160 (66.95)		917 (71.75)	
		Other drug	352 (33.88)		83 (34.73)		435 (34.04)	
	**SUD diagnoses**							
		Alcohol	68 (5.41)		18 (6.08)		86 (5.54)	
		Cannabis	67 (5.33)		11 (3.72)		78 (5.03)	
		Opioid	—		—		26 (1.68)	
		Stimulant	20 (1.59)		5 (1.69)		25 (1.61)	
		Other drug	31 (2.47)		9 (3.04)		40 (2.58)	
	Any drug overdose		—		—		6 (0.39)	
	Prescribed buprenorphine		31 (2.47)		5 (1.69)		36 (2.32)	
**Engagement in SUD-related care, n (%)**								
	Mental health specialty		138 (10.99)		35 (11.82)		173 (11.15)	
	Addictions specialty		—		—		—	
	Integrated mental health in primary care		—		—		7 (0.45)	

^a^To avoid potential identification, numbers are masked in both clinics if the N in either clinic is <5. Total Ns are masked if <5.

^b^Defined as bipolar spectrum disorders, schizophrenia spectrum disorders, and other psychosis, consistent with previous studies [63].

^c^Defined as the Patient Health Questionnaire-2 score of ≥3 [64]. Missing data for 386 patients (clinic A: n=311; clinic B: n=75).

^d^SUD: substance use disorder.

^e^A high-scoring screen was defined as a score of ≥7 on the Alcohol Use Disorders Identification Test [65], a score of 4 on the Single-Item Screen for Cannabis [46], and a score >0 for other drugs on the Single-Item Screen for Drugs [47,48]. Screening data were missing for 274 patients (clinic A: n=217; clinic B: n=57).

#### Reach, Fidelity, and Treatment Engagement

**Figure 5:**
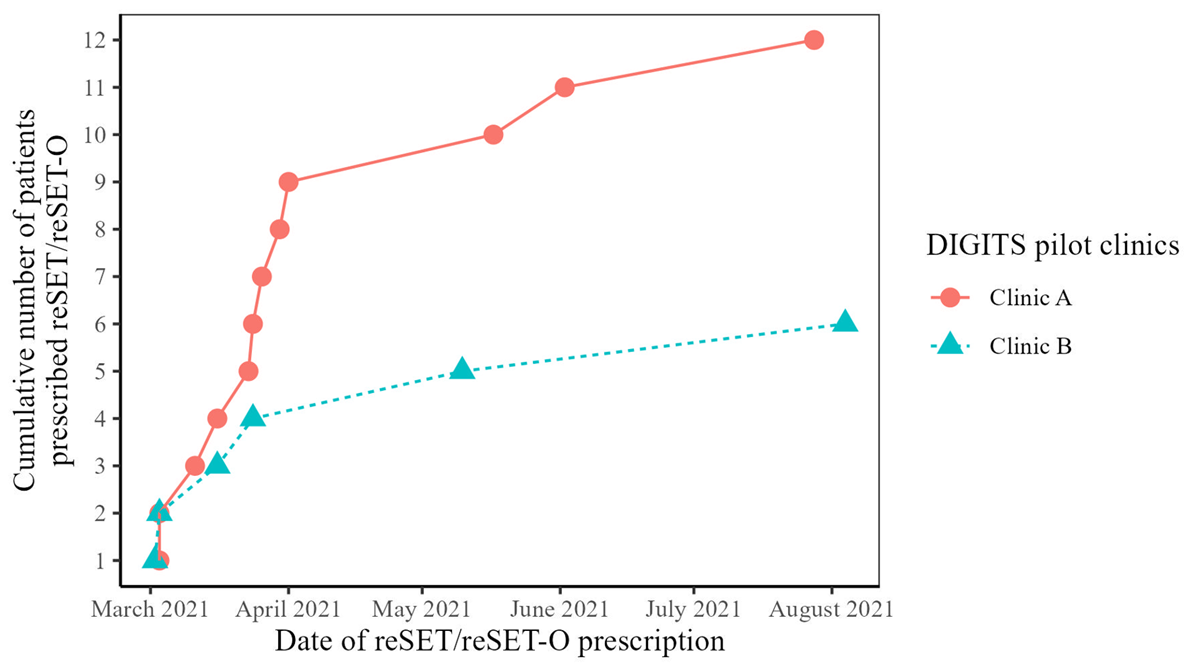
Digital therapeutic prescriptions over time. The Digital Therapeutics for Opioids and Other Substance Use Disorders (DIGITS) pilot study used mixed methods to evaluate the implementation of 2 digital therapeutics (reSET and reSET-O) for substance use disorders in 2 primary care clinics in the same integrated health system in Washington State.

Most prescriptions occurred in the first month of the pilot (Figure 5). The main outcome measures for the main analytic cohort and patients prescribed reSET and reSET-O are presented in Table 3. Clinicians prescribed the digital therapeutics to 0.9% (14/1552) of patients in the main analytic cohort and 18 patients in total (clinic A: n=12, 67%; clinic B: n=6, 33%). Notably, 4 (22%) patients who were prescribed the digital therapeutics did not meet the eligibility criteria for the main analytic cohort; 3 (17%) patients did not have a high-scoring drug screen, and 1 (6%) did not visit a pilot clinic during the accrual period (they may have been referred from another clinic). Characteristics of the prescribed patients are included in Multimedia Appendix 1.

**Table 3:** Digital therapeutic reach and fidelity.

Outcome			Main analytic cohort (n=1552)^a^						Prescribed cohort (n=18)^b^				
			Clinic A (n=1256): standard implementation, practice facilitation, and health coaching		Clinic B (n=296): standard implementation and health coaching		Total (n=1552)		Clinic A (n=12): standard implementation, practice facilitation, and health coaching		Clinic B (n=6): standard implementation and health coaching		Total (n=18)
**Reach, n (%)**													
	Prescribed (reach-2)	8 (0.64)		6 (2.03)		14 (0.9)		12 (100)		6 (100)		18 (100)	
	Downloaded and activated (reach-3)	6 (0.48)		1 (0.34)		7 (0.45)		9 (75)		1 (16.67)		10 (55.56)	
	≥1 module used (reach)	4 (0.32)		1 (0.34)		5 (0.32)		7 (58.33)		1 (16.67)		8 (44.44)	
**Fidelity (wk), mean (SD)**													
	At least 1 module/week (fidelity-2)	0.01 (0.35)		0.00 (0.06)		0.01 (0.31)		3.17 (4.61)		0.17 (0.41)		2.17 (3.99)	
	At least 4 modules/week (fidelity-3)	0.00 (0.09)		0.00 (0.06)		0.00 (0.08)		1.33 (2.96)		0.17 (0.41)		0.94 (2.46)	
	At least 4 modules/week+monthly visit (fidelity)	0.00 (0.06)		0.00 (0.06)		0.00 (0.06)		1.00 (2.59)		0.17 (0.41)		0.72 (2.14)	
**Treatment engagement (mo), mean (SD)**													
	At least 1 SUD-related visit/month	0.00 (0.13)		0.01 (0.12)		0.01 (0.13)		1.17 (1.27)		0.33 (0.82)		0.89 (1.18)	

^a^The main analytic cohort (our predefined study eligibility criteria) consisted of patients aged ≥18 years who had a primary care visit at clinic A or B during the accrual period (from February 23 to August 6, 2021) and had a high-scoring screen for drug use the day of the visit or at any time in the prior year. A high-scoring screen was defined as a score of ≥7 on the Alcohol Use Disorders Identification Test [66], a score of 4 on the Single-Item Screen for Cannabis [46], and a score >0 for other drugs on the Single-Item Screen for Drugs [47,48].

^b^Four of 18 patients prescribed the digital therapeutics did not meet the preestablished eligibility criteria for the main analytic cohort.

**Figure 6:**
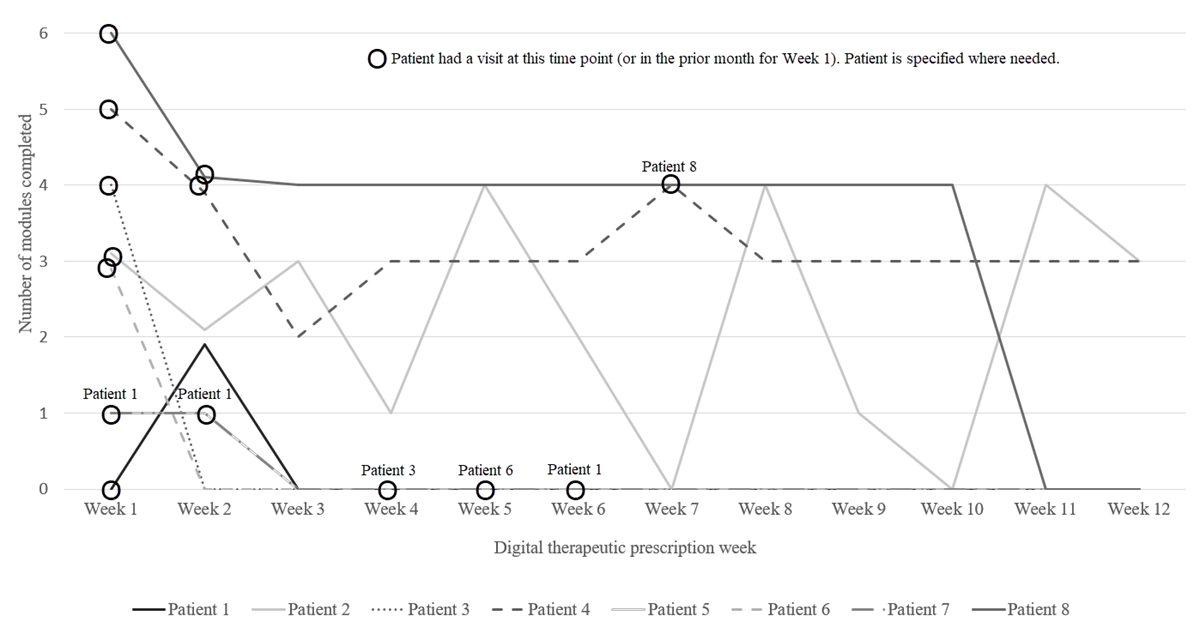
Weekly module completion and treatment engagement for patients who activated the digital therapeutic and completed at least 1 module (n=8).^a^Modules are core content sections within the digital therapeutics. The digital therapeutic vendor recommended patients complete four modules each week.​ Treatment engagement refers to patient engagement in substance use disorder-related visits with a clinician. Data for patient 5 and patient 3 are entirely overlapping. Patient 7 did not have a visit during the 12-week digital therapeutic prescription or in the month prior. Two patients activated their prescription but did not complete any modules and are therefore excluded from the visualization. Both of these patients had a visit prior to week 1 and during week 4.

A total of 10 patients (56% of prescribed patients; clinic A: n=9, 90%; clinic B: n=1, 10%) downloaded the app and activated their prescriptions, and 8 (44% of prescribed patients; clinic A: n=7, 88%; clinic B: n=1, 12%) completed at least 1 module. Patients who used the app completed 1 (SD 1.6) module per week on average. While 4 (22% of prescribed patients) patients completed the recommended 4 modules per week at least once for a total of 17 patient weeks, 2 (11% of prescribed patients) patients completed all 31 core modules. Only 1 (6%) patient ever met our definition of the highest level of fidelity (completing 4 modules per week and monthly SUD visits), and they did so persistently for a total of 10 weeks. A total of 9 (50%) patients who were prescribed the app had any SUD-related visit during their prescription period, and 2 (11%) patients completed monthly visits for all 3 months. In Figure 6, weekly module completion and treatment engagement are visualized over time for patients who activated the digital therapeutic and completed at least 1 module (8/18, 44% of prescribed patients). Regarding adoption, as above, all 3 out of the 3 LICWSs working at the clinics during the pilot accrual period were trained to prescribe the digital therapeutics and intended to use them with patients. However, clinical leaders in the delivery system decided to postpone the training of non-LICSW clinicians (due to concerns about the time burden of training), so no other clinician types adopted the digital therapeutic during the pilot accrual period (not shown in the table).

### Qualitative Results From Formative Evaluation

Findings from the formative evaluation provided insights into the barriers and facilitators encountered when connecting eligible patients to the digital therapeutics. The barriers included understaffing of LICSWs at our pilot clinics, understaffing of LICSWs across KPWA at large (requiring LICSWs at the pilot clinics to provide coverage for clinics without LICSWs), limited capacity, competing priorities, and turnover of LICSWs trained to offer the digital therapeutics. LICSWs reported working with unprecedented numbers of patients with suicidal ideation during the trial (emerging evidence suggests suicidal ideation may have increased during the COVID-19 pandemic) [67,68]. These visits took priority over all nonurgent visits. The first LICSW from clinic A told us that at one point, they were only following up on referrals labeled “urgent” in the EHR. When LICSWs did receive urgent referrals related to SUD, those patients often needed a higher level of support than digital therapeutics could provide. Additional barriers for LICSWs included difficulty making PCPs aware of reSET and reSET-O as options for treatment (in the context of busy, constantly evolving primary care clinics); technical challenges with the app; and difficulty with the novel and logistically complex processes required to offer apps (eg, multiple steps to connect patients to the apps, unfamiliar tools, and workflows in the EHR [including routing prescriptions to a medical doctor]). LICSWs shared that some patients who were interested in digital therapeutics did not want monthly SUD-related visits. If a patient had a follow-up appointment scheduled but canceled, the LICSW generally did not have time to try to reschedule. This was a barrier to completing monthly SUD-related visits that were required while using the apps.

During the pilot, a key facilitator was high engagement from clinical leaders and LICSWs. Additional facilitators included the ability to program fillable templates into the EHR and the perception that the digital therapeutics for SUD could meet patient needs. One of the goals of a formative evaluation is to identify modifications and adaptations to improve implementation. In response to the feedback from LICSWs and clinical leaders, we simplified the training materials and created a way to track patients who were offered reSET or reSET-O but were not ready to try them.

While we cannot say for certain why prescription rates were initially high and then leveled off, meeting notes provide some insights to explain patterns over time. After programmers resolved technical problems with the electronic medical record and the vendor’s dashboard (see the *Data Collection and Analysis* section), clinicians prescribed the digital therapeutic to 13 patients in a 6-week period. After this point, prescribing slowed considerably, with only 5 additional prescriptions for the rest of the pilot (about 13 weeks). Meeting notes show staffing shortages and turnover, which limited the capacity of the LICSWs at our pilot clinic, worsened throughout the pilot course. For example, 1 LICSW reported that when a cross-clinic coverage program (designed to provide services to clinics without LICSWs) was launched on March 1, 2021, the covering LICSW was responsible for 5 clinics; by April 26, 2021, the covering LICSW was responsible for 10 clinics. In addition, it is possible that clinician excitement about a new resource boosted prescription rates early in the trial. It is also possible that at the beginning of the pilot, clinicians prescribed to patients they had already worked with and who they thought could benefit from reSET or reSET-O but then did not subsequently identify many additional patients to prescribe the apps to.

## Discussion

### Principal Findings

This study presents detailed findings from a real-world digital therapeutic implementation initiative in primary care. We trained 3 LICSWs in 2 primary care clinics to connect patients with SUD to prescription digital therapeutics. We successfully delivered standard implementation processes and PF support. A total of 18 patients were prescribed the app in a 6-month period, about half of whom downloaded and activated it and completed at least 1 module (activated: 10/18, 56%; completed at least 1 module: 8/18, 44%). Reach was low overall and limited by staffing shortages, limited LICSW capacity, and LICSW attrition during the pilot. HC was offered to a few patients but was not used during the pilot.

There are limited data on the implementation of digital therapeutics for SUD in real-world primary care settings (where clinicians identify patients and offer the apps as part of routine care delivery) to which we can compare our results. Our study sample had low SUD treatment engagement overall, with baseline data showing only 11.66% (181/1552) of patients had received any care for SUD in the prior 2 years. Novel treatments may be able to expand the number of people who obtain SUD treatment but may not be able to overcome the numerous preexisting challenges related to connecting patients to SUD treatments.

The same digital therapeutics implemented in our study have been deployed in addiction treatment settings, and studies by the vendor suggested high engagement with the apps (percentage of patients who had completed at least 1 module completing 4 modules per week ranged from 36% to 73% [18], compared with our 13% to 25%). It may be possible that patients in addiction treatment settings are more likely to engage in treatment with digital therapeutics, especially because patients in these settings are actively seeking treatment and often see clinicians weekly or more frequently. Additional research is needed to understand how setting influences digital therapeutic reach and fidelity; for instance, prior reviews of health care system factors that influence the uptake of digital therapeutics did not study health care settings as a factor [24].

Another primary care–based implementation study of an app-based treatment for SUD connected 8.3% of eligible patients to the app (compared with our 0.9%), but there are critical differences between the 2 studies [69]. First, Quanbeck et al [69] did not use a prescription digital therapeutic. The requirement for prescription approval from a medical doctor complicated our implementation, and LICSWs in our study identified this as a barrier to offering the apps. Second, their implementation effort included a preimplementation phase that included PF, workflow planning, and PDSA cycles. If we were to compare our studies directly, this pilot is somewhat like their preimplementation phase. Third, in the other study, app delivery deviated from care as usual; a site coordinator was available in person to enroll patients, help set up the app, and train patients on how to use the app. That research study also provided patients with a phone if needed (up to 100 phones per clinic). During this pilot study, enrollment activities were enacted by LICSW clinicians in primary care during health care visits, and most LICSW visits were virtual (rather than in person). This likely limited the opportunities LICSWs had to provide technical support for downloading or using the digital therapeutics. Fourth, the analytic samples of the studies differed. The analytic sample of the other study included patients with a documented SUD diagnosis, making their inclusion criteria narrower than ours; we used an analytic sample that included all patients who screened positive for cannabis or drug use because SUDs are vastly underidentified in primary care. Only 8.31% (129/1552) of patients in our sample had a documented SUD diagnosis (excluding AUD since the apps we implemented were not designed to treat AUD as a sole diagnosis). Notably, 27.78% (5/18) of patients who were prescribed the digital therapeutics had a documented SUD diagnosis (excluding AUD). It may be possible that patients with a diagnosis are more likely to be offered SUD treatments; alternatively, SUDs may be more likely to be documented when there is increased availability for treatment options.

Low reach in our study may be explained in part by our broad eligibility criteria. The extremely high rate of underdiagnosis of SUD in health care systems makes it difficult to accurately measure the true number of eligible patients. To account for this, we opted to include all patients who had a high-scoring drug use screen in our study, which we knew would increase the size of our main analytic cohort to include more patients who did not meet the criteria for SUD (which was an eligibility requirement for the digital therapeutics). In addition, although all the patients in our main analytic cohort had a visit during the accrual period, they most likely did not interact with the LICSW trained to offer the apps, as they could have visited a PCP but not the LICSW. Finally, 63.21% (981/1552) of patients were deemed eligible for the analyses based on self-reported daily or almost daily cannabis use without other drug use. While research has shown this single-item screening question to perform well in identifying patients with cannabis use disorder [47], it is possible that not everyone who reported daily cannabis use had a documented SUD or saw their cannabis use as needing intervention. In addition, recreational cannabis use was legal in Washington State during the pilot study. Previous research suggests recreational legalization and social acceptance of cannabis reduce the perceived risk of its use and decrease engagement in treatment for cannabis use disorder [70,71].

In addition to our study population being potentially too broad, the fact that 3 patients who were prescribed the app did not have a high-scoring drug screen indicates an additional challenge with accurate cohort identification. It is possible that patients did not answer accurately about their level of drug use on the screen or that patients or LICSWs identified a benefit to treatment for less-frequent drug use.

The percentage of patients who activated their app prescription (about 50%) is likely an estimate that can be expected in future implementations because our activation rate is similar to what was found in a health care claims analysis conducted by the app vendor [19]. Because of the small number of patients (10/18, 55.56%) in our study who activated the app, we cannot draw conclusions regarding app engagement. Previous research has shown low engagement with health-focused apps over time [72], but a larger study in real-world settings is needed to provide baseline expectations for how much primary care patients will use SUD apps. Similarly, we cannot draw conclusions regarding the uptake of HC because only 2 of 4 (50%) patients eligible for HC were reached by the HC MA. It is possible that patients interested in app-based treatments are not interested in human coaching by phone, but more research is needed to understand patient preferences in this regard.

Although we encountered implementation challenges, most notably staffing strain, we had some implementation successes. We had high engagement with PF, and LICSWs provided positive feedback about PF and HC support. We also refined the PF and HC processes in preparation for the larger DIGITS Trial. We simplified training materials, created a “huddle card” for LICSWs to advertise reSET and reSET-O to PCPs in their clinics, created templated text that clinicians could use to send messages to patients via the patient portal if they did not activate the app, dropped a requirement that patients must be paneled at the same clinic where the LICSW was assigned, and created a way to track patients who were offered the digital therapeutics but not ready to try them (so clinicians could follow up with these patients). Our formative evaluation was very effective in identifying the barriers and facilitators to implementation.

### Strengths and Limitations

This pilot study has several strengths. Outcomes were prespecified and grounded in implementation science methodologies. We also used a pragmatic design and implemented digital therapeutics in a real-world health system, which makes our findings more relevant than trials with a selective patient population.

This study has several limitations. Our pilot was impacted by the challenges related to the COVID-19 pandemic and its aftermath, including staff turnover and potential population-level changes in mental health care needs. Our study sample is not very racially or ethnically diverse, which may limit the applicability of our findings. Our measurement of fidelity relied on EHR data and insurance claims data. While claims data can detect prior SUD treatment that is reimbursed by KPWA, it cannot detect treatment that was not reimbursed. In addition, we did not capture downloads or activations that occurred after our predefined outcomes assessment period, so delayed app use could have been missed. We also did not collect data on patient access to adequate technology to use the digital therapeutics (eg, smartphone ownership) and therefore do not know if or to what extent this was a barrier to reach or fidelity. This pilot study only involved 2 clinics and was not randomized. The primary DIGITS Trial will be randomized and should illuminate the extent to which PF and HC improve the implementation of digital therapeutics.

### Conclusions

Connecting the large number of patients in primary care who could benefit from digital therapeutics for SUD to these app-based treatments is challenging, especially when understaffing, high turnover, and competing priorities are present.

## Appendix

Multimedia Appendix 1 Baseline characteristics of the patients who were prescribed the digital therapeutics (n=18).

## Abbreviations

AUD: alcohol use disorder
DIGITS: Digital Therapeutics for Opioids and Other Substance Use Disorders
EHR: electronic health record
FDA: Food and Drug Administration
HC: health coaching
KPWA: Kaiser Permanente Washington
LICSW: licensed independent clinical social worker
MA: medical assistant
PCP: primary care provider
PDSA: plan-do-study-act
PF: practice facilitation
SUD: substance use disorder
