# Supplementary material for: Prescription Digital Therapeutics for Substance Use Disorder in Primary Care: Mixed Methods Evaluation of a Pilot Implementation Study
Source: JMIR Form Res. 2024 Sep 2;8:e59088. doi: 10.2196/59088 (PMC11406110; doi:10.2196/59088)
Supplement: Multimedia Appendix 1 [file formative_v8i1e59088_app1.docx]

| Demographics | n (%) |
| --- | --- |
| Age in years (mean ± SD) | 30.9 (8.3) |
| Age in years | |
| 18-24 | 5 (27.8) |
| 25-34 | 8 (44.4) |
| 35-44 | 5 (27.8) |
| Sex | |
| Female | 9 (50.0) |
| Race | |
| Asian | --^c^ |
| Black or African American | 0 (0.0) |
| American Indian / Alaska Native | -- ^c^ |
| Native Hawaiian or Pacific Islander | 0 (0.0) |
| White | 12 (66.7) |
| Multiple race | 0 (0.0) |
| Other race | 0 (0.0) |
| Missing race data | -- ^c^ |
| Ethnicity | |
| Hispanic | -- ^c^ |
| Non-Hispanic | -- ^c^ |
| Missing ethnicity data | 8 (44.4) |
| Rurality of residence (census tract) | |
| Urban | 18 (100.0) |
| Insurance type | |
| Medicaid | -- ^c^ |
| Medicare | 0 (0.0) |
| State subsidized | -- ^c^ |
| Private Pay, Self-funded, High Deductible and Basic Health | -- ^c^ |
| Commercial | 11 (61.1) |
| One year or more of insurance enrollment | 11 (61.1) |
| Health status |  |
| Mental health diagnoses | |
| Anxiety | 13 (72.2) |
| Depression | 12 (66.7) |
| Serious mental illness^d^ | -- ^c^ |
| Positive screen for depression^e^ | 12 (85.7) |
| Any emergency visits | -- ^c^ |
| Any hospitalization | -- ^c^ |
| SUD-related characteristics |  |
| High-scoring drug use screens^f^ | |
| Alcohol | 8 (57.1) |
| Cannabis | -- ^c^ |
| Other drug | 9 (64.3) |
| SUD diagnoses | |
| Alcohol | 8 (44.4) |
| Cannabis | -- ^c^ |
| Opioid | -- ^c^ |
| Stimulant | -- ^c^ |
| Other drug | 5 (27.8) |
| Any drug overdose | -- ^c^ |
| Prescribed buprenorphine | -- ^c^ |
| Engagement in SUD-related care | |
| Mental health specialty | 7 (38.9) |
| Addictions specialty | 0 (0.0) |
| Integrated mental health in primary care | -- ^c^ |

aThe time-period for constructing baseline clinical characteristics was from two years before until the day before the qualifying visit.b Age was measured on the date of the qualifying visit. Sex (binary male/female), race, and ethnicity were from the time of the data pull to get the most current data. Insurance and address (used to classify rurality/urbanity using the 2010 rural-urban commuting area codes and 2010 Census Tract geocoding) were measured on the date of or in the month before the qualifying visit, depending on data availability.

bPrescription date was used as a proxy qualifying visit date for 4 patients missing a qualifying visit).

cTo avoid potential identification, we masked Ns less than five and Ns of mutually exclusive categories that could be used to deduce Ns less than five.

dDefined as bipolar spectrum disorders, schizophrenia spectrum disorders, and other psychosis, consistent with previous studies.[62]

eDefined as PHQ2 score greater than or equal to three.[63] Missing data for 4 patients.

fA high-scoring screen was defined as a score of seven or more on the Alcohol Use Disorders Identification Test (AUDIT-C),[52] a score of four on the Single Item Screen for Cannabis (SIS-C)[46] and a score greater than zero for other drugs on the Single Item Screen for Drugs (SIS-D).[47,48]
